# Undifferentiated hepatic carcinoma with osteoclast-like giant cells: A case report and literature review

**DOI:** 10.3389/fonc.2022.1018617

**Published:** 2023-01-09

**Authors:** Yujiao Deng, Ya Wang, Yan Zhang, Na Yang, Xingli Ji, Bing Wu

**Affiliations:** ^1^ Department of Radiology, West China Hospital, Sichuan University, Chengdu, China; ^2^ Department of Radiology, Chengdu Fifth People’s Hospital, Chengdu, China; ^3^ Department of Pathology, Chengdu Fifth People’s Hospital, Chengdu, China

**Keywords:** osteoclast-like giant cell, undifferentiated hepatic carcinoma, diagnosis, treatment, prognosis

## Abstract

Osteoclast-like giant cell tumor (OGCT) is a common bone tumor, occasionally observed in some extraosseous organs, but rarely involving the digestive system, especially the liver. Previously reported osteoclast-like giant cell carcinoma of the liver often coexists with sarcomatoid or hepatocellular carcinoma. Undifferentiated liver tumors with osteoclast-like giant cells (OGCs) are extremely rare. Due to its rarity, there is no consensus for diagnosis and treatment of undifferentiated liver tumors with OGCs. Definitive diagnosis comes from surgery, so there is often a long delay in diagnosis following the occurrence of symptoms. This case describes an extremely rare case of an undifferentiated liver tumor with OGCs in detail. It also summarizes the previously published cases based on liver tumors with OGCs from August 1980 to June 2021, providing extensive evidence to improve preoperative diagnosis and management options.

## 1 Introduction

Giant cell tumor of bone (GCTB) is a benign mesenchymal tumor, affecting mostly long bones. It usually develops in young adults (20–40 years old). Histologically, it is mainly composed of numerous osteoclast-like giant cells (OGCs) histologically (1). Notably this type of tumor, as reported, was also found at several extraskeletal sites (2). Among the digestive system organs, the pancreas was designated as the most vulnerable to developing this pathology (3). The occurrence of undifferentiated liver tumors manifesting as OGCs is an extremely rare event. According to the latest edition of the WHO classification of digestive system tumors, primary hepatic undifferentiated carcinoma meets the diagnostic criteria for rare liver tumors (4). A total of 18 instances associated with hepatic tumors with cell components of OGCs have been described since the first case was reported in 1980 (5). However, less than three cases are undifferentiated liver tumors, with OGCs among these instances. Due to limited information, clinical manifestations and imaging features cannot be well understood and summarized. Misdiagnosis and delayed diagnosis might easily happen, leading to a poor prognosis. Here, we present a rare case of undifferentiated liver tumor with OGCs and review previously published cases of liver tumor with OGCs from August 1980 to June 2021. We discuss the epidemiology, clinical manifestations, imaging features, pathological features, differential diagnosis, treatments, and prognosis of OGCT in detail, which is to collect more information systematically for disease decision-making.

## 2 Case presentation

### 2.1 History and examination

A 68-year-old man presented with intermittent right upper quadrant (RUQ) abdominal pain for half a year. Additionally, a temperature of up to 38.3°C occurred more than a month ago with no apparent cause. Upon arrival, he admitted having diabetes for three years, treated with metformin and glimepiride. His vital signs were stable upon initial evaluation. There is no evidence of a family history of cancer. The patient had a long history of alcohol abuse (daily consumption of >250 g alcohol over the past 30 years). Physical examination revealed no noteworthy findings other than abdominal pain. Tumor markers revealed elevated alpha-fetoprotein (AFP) levels (7.03 ng/ml) and des-gamma-carboxy prothrombin (DCP) levels (33.0 mAU/ml). A complete hemogram showed elevated RDW-CV and AMC levels, and decreased RBC, HGB, HCT, and LY% levels. Liver function tests showed elevated ALT, AST, GGT, and glucose levels and decreased TP and albumin levels. Renal function tests showed elevated serum cystatin C levels. A complete hemogram, liver function, and renal function tests of the patient are presented in **Table 1**. Laboratory tests also showed positive E antibody and core antibody.

**Table 1:** Blood tests.

Items	Result	Unit of measurement	Reference value	Mark
Red blood cell count, RBC	2.70	×10^12^/L	4.3–5.8	↓
Hemoglobin, HGB	78	g/L	130–175	↓
Hematocrit in blood, HCT	0.24	L/L	0.40–0.50	↓
Red blood cell distribution width coefficient of variation, RDW-CV	15	%	11.5–14.5	↑
Lymphocyte percentage, LY%	16.2	%	20–50	↓
Absolute monocyte Count, AMC	0.64	×10^9^/L	0.1–0.6	↑
Alanine aminotransferase, ALT	74	IU/L	<50	↑
Aspartate aminotransferase, AST	41	IU/L	<40	↑
Gamma-Glutamyl transpeptidase, GGT	72	IU/L	<60	↑
Total protein, TP	63.1	g/L	65.0–85.0	↓
Albumin	34.9	g/L	40–55	↓
Glucose	6.13	mmol/L	3.9–5.9	↑
Serum cystatin C	1.13	mg/L	0.51–1.09	↑

### 2.2 Abdominal imaging findings

**Figure 1:**
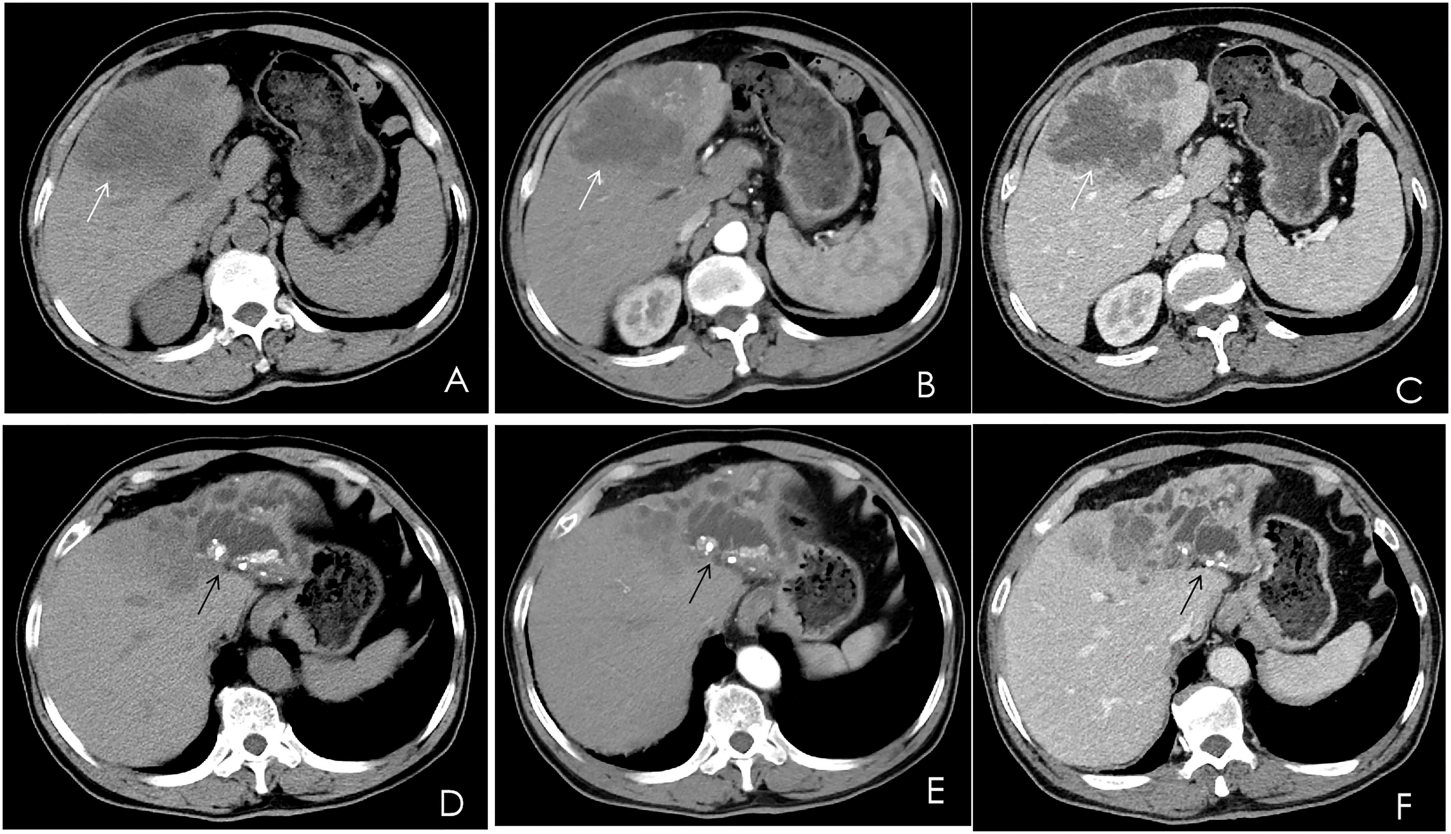
Multiphase contrast-enhanced CT of the upper abdomen, axial view. **(A)** A noncontrast CT showed multiple low-density masses and nodular mixed-density shadows in the left lobe and part of the anterior right lobe of the liver (white arrow). **(B)** The arterial phase showed heterogeneous ring enhancement (white arrow). **(C)** The venous phase showed continuous enhancement (white arrow). **(D–F)** The left branch of the intrahepatic bile duct and its branches were dilated, and high-density nodular shadows were seen in the liver (black arrow).

A contrast-enhanced computed tomography (CT) scan of the upper abdomen displayed multiple slightly low-density masses and mixed-density nodules in the left lobe and the anterior segment of the right lobe. The largest mass was measured at 11.2 × 8.5 cm from an axial view. Lesions showed ring enhancement in the arterial phase and continuous enhancement in the portal phase. The sagittal part of the portal vein was infiltrated. Dilatation of the left intrahepatic bile duct combined with bile duct stones was observed. In addition, there were enlarged lymph nodes in the hepatic portal, portacaval space, and around the abdominal aorta (**Figure 1**).

### 2.3 Surgery

A preoperative diagnosis of cholangiocarcinoma was made. Resection of the left lateral lobe, left medial lobe, right anterior lobe, and caudate lobe of the liver, hilar cholangioplasty, and cholecystectomy were performed to remove the whole mass after two days of admission, which appeared to have a complete tumor margin, a yellowish cross-section, and dilated partial bile ducts with stones inside. A lymphadenectomy was not performed. The operative time was 228 min. The blood loss was 500 cc, and a perioperative blood transfusion was not required. Surgical samples were taken and sent for pathological examination. There were no immediate postoperative complications.

### 2.4 Pathological findings

**Figure 2:**
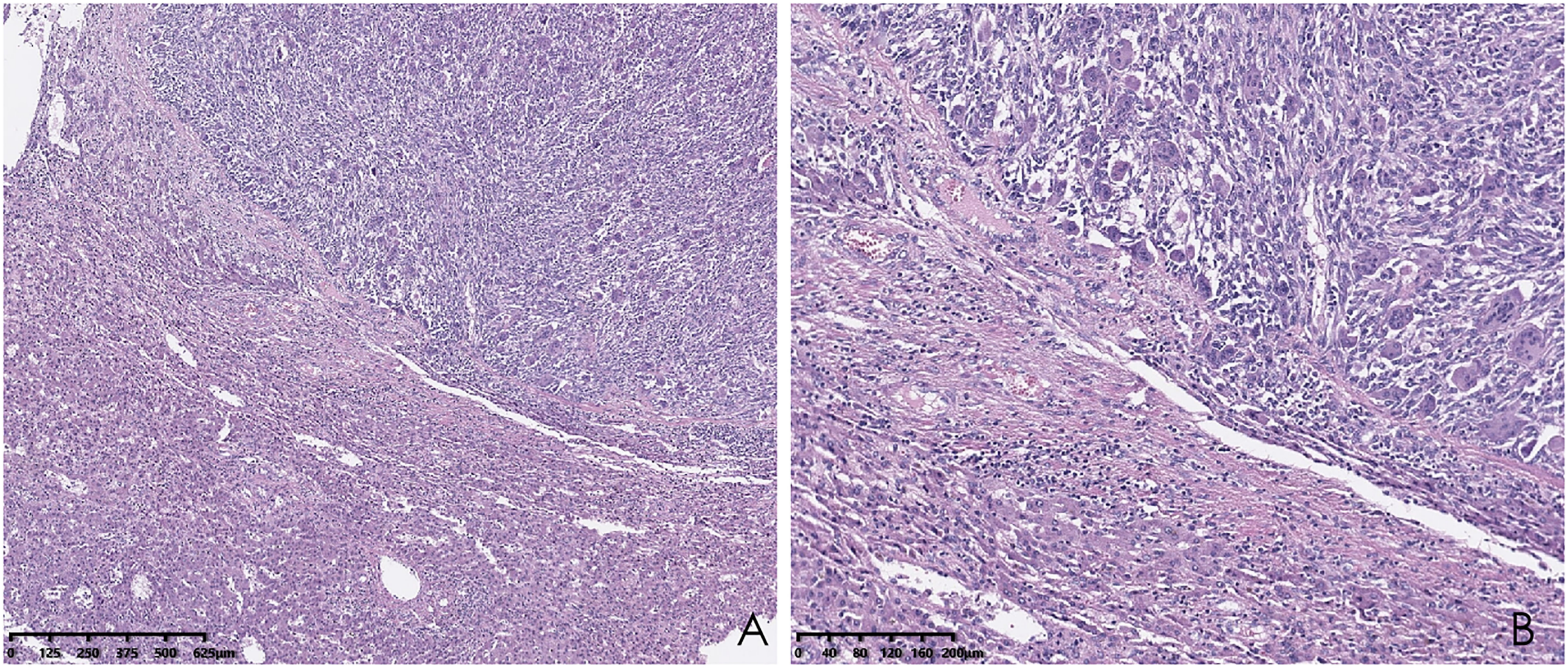
Hematoxylin and eosin (HE) staining of the liver biopsy sample shows osteoclast-like giant cells and neoplastic spindle-shaped cells. (**A**; hematoxylin and eosin, ×40); higher magnification of the destroyed liver structure lined by osteoclast-like giant cells and neoplastic spindle-shaped cells (**B**; hematoxylin and eosin, ×100).

**Figure 3:**
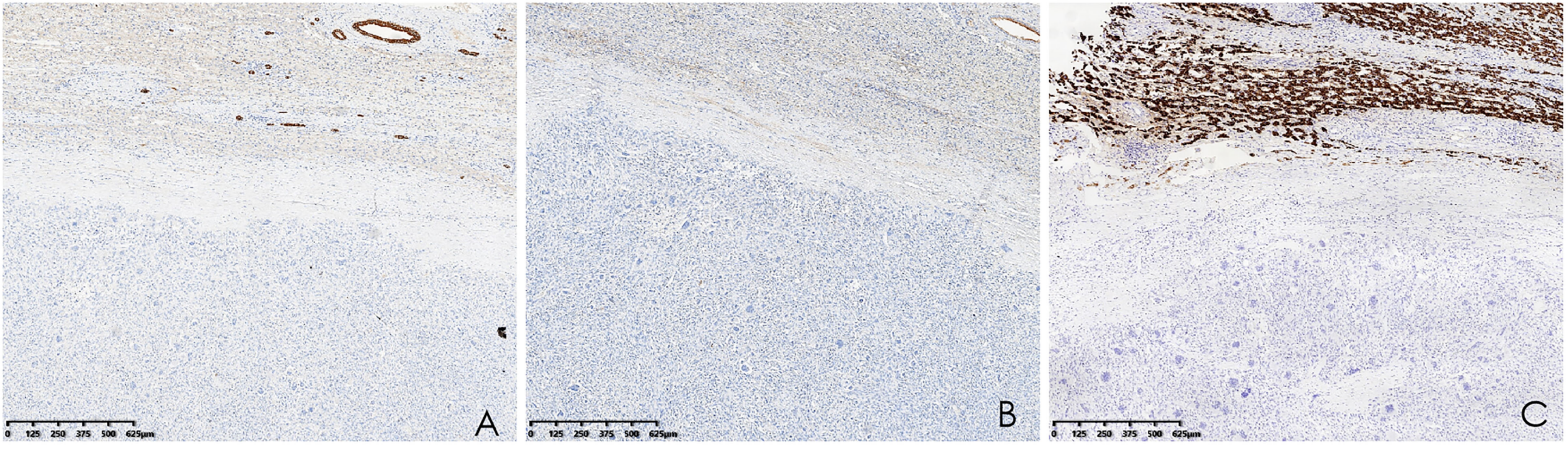
**(A–C)** Immunohistochemistry showed that CK (Pan), EMA, and Hepa were negative (hematoxylin and eosin, ×40).

**Figure 4:**
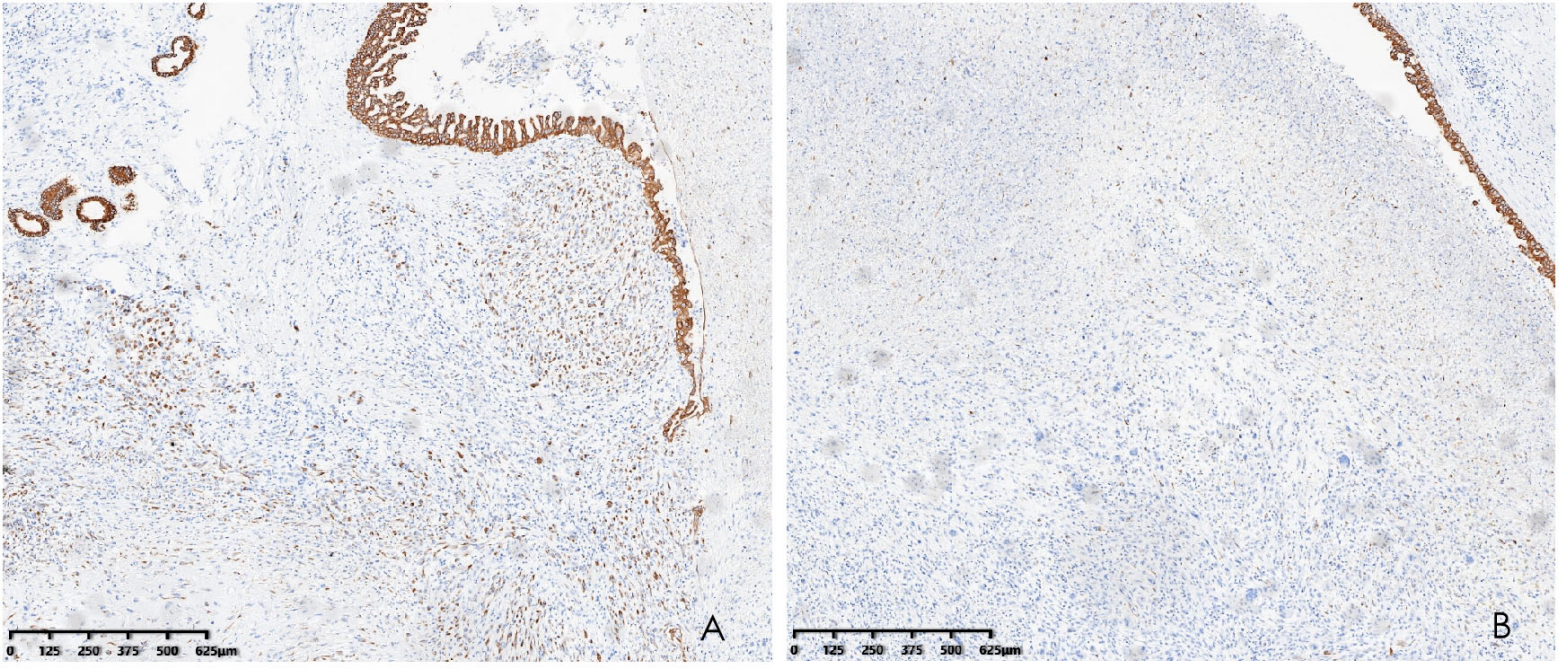
Immunohistochemistry demonstrated CK8/18 positivity in the tumor **(A)** and CK8/18 negativity for osteoclasts **(B)** (hematoxylin and eosin, ×40).

Histopathological analysis revealed that tumors were rich in osteoclast-like giant cells and neoplastic spindle-shaped cells (**Figures 2A, B**). Complete hepatic-lobule-like structures, edema of hepatocytes, intrahepatic cholestasis, lymphocyte, and plasma cell infiltration in the portal area were observed in the non-tumorous liver parenchyma of the resected specimen. Immunohistochemical staining showed that tumor cells were negative for EMA, Hepa, CK (Pan) (**Figures 3A–C**), desmin, CD34, S-100, SATB2, P63, P16, GS, GPC3, and CAM5.2, but positive for SMA, P53, Ki-67 (30%), ACT, CK8/18 (**Figures 4A, B**), and vimentin. PCR and Sanger sequencing: no H3F3A gene mutation was detected. Combined with histopathology and immunohistochemistry, the diagnosis of undifferentiated carcinoma with osteoclast-like giant cells of the liver was considered. The possibility of metastatic tumors was excluded by a comprehensive clinical examination.

### 2.5 Postoperative course

The postoperative situation of the patient was stable, and he was discharged on the 12th day. At the first follow-up two months after hospital discharge, a chest CT showed that the lymph nodes in the mediastinum and right diaphragmatic corner were significantly increased. The patients and their families refused gene tests and agreed to perform GA chemotherapy was temporarily performed (gemcitabine 1.5 g d1; d8 + albumin paclitaxel 200 mg d1; d8). At the second follow-up five months after hospital discharge, metastases were found on CT scans of the head and chest. The patient and his family still refused the gene test and chose to undergo GP chemotherapy (gemcitabine 1,600 mg d1, 8 ivgtt + cisplatin 40 mg d1, 8; Q3w) and radiotherapy for the head. The current vital signs of the patient are stable. The patient is alive after 5-month follow-up.

## 3 Discussion

### 3.1 Epidemiology

Munoz et al. (5) first recorded an instance of OCGT of the liver, and some additional patients suffering from this rare tumor at the same site have been reported since then. These previously reported carcinomas were classified as tumors related to hepatocellular carcinomas, tumors related to cholangiocarcinomas or cystadenocarcinomas of the liver, tumors related to sarcomatous tumors, and undifferentiated tumors that were not related to conventional carcinomas, as presented in this case (6). Undifferentiated primary carcinoma of the liver itself is an uncommon type of cancer. The presence of osteoclast-like giant cells in undifferentiated carcinoma of the liver rarely occurs, classified as T3N0M0 and Stage III according to the third English edition of the Japanese classification of liver cancer. Only a few previous reports of liver tumors with giant cells resembling osteoclasts have been reported, so little is known about their biological behavior and clinical course.

It is well known that blood serum levels of PIVKA-II and AFP are useful indicators for the diagnosis of HCC. Suehiro et al. confirmed that a poorer prognosis is associated with a high level of serum PIVKA-II (7). In this case, the serum PIVKA-II level was elevated in this patient.

### 3.2 Clinical presentation

Case reports associated with hepatic tumors with OCGs, published from August 1980 to June 2021, were searched through the Biomedical Literature Database (PubMed). Clinical manifestations of hepatic tumors with OCGs are listed in **Table 2**. As evident from the 18 reported cases, the disease is more common in males (12, 66.7%), and its onset age ranges from 37 to 87 years old (average age: 63.7 years old), mostly affecting the left lobe of the liver. Several clinical symptoms have been reported at presentation, including abdominal pain, nausea, abdominal distension, fever, weight loss, and so on. Some cases were associated with cirrhosis (7, 38.9%) and hepatitis (6, 33.3%). AFP is a specific tumor marker for HCC, and the level of the tumor marker was elevated in four patients. As for previous history, most patients suffered from hypertension and diabetes (3, 5, 6, 8–22).

**Table 2:** Clinical manifestation of hepatic tumor with OCGTs.

Study	Year	Sex	Age	Clinical manifestation	Tumor location	Tumor size	Hepatic disease	Cirrhosis	Previous history	AFP
Phillip a. Munoz (5)	1980	man	87	lethargy, anorexia, and fluid retention	right lobe of the liver	11 cm in diameter	portal hypertension and chronic hepatic failure	macronodular cirrhosis of the liver	insulin-dependent diabetes; resection of Grade I1 papillary transitional cell carcinoma of the urinary bladder	normal
Hlroyukl Kuwano (8)	1984	man	54	general fatigue with slight fever, night sweating, weight loss	right lobe of the liver	6x5x12 cm	type b hepatilis	hepatic cirrhosis	negative	206.3 ng/ml
Salvatore Andreola (9)	1985	man	71	eneral fatigue and upper abdominal discomfort	right lobe of the liver	12 cm in diameter	hepatitisB	hepatic cirrhosis	chronic alcoholism	normal
Yutaka Hori (10)	1987	male	66	hematemesis, nausea, epigastric dullness, abdominal pain	inferior surface of the liver	unknown	negative	negative	negative	normal
Daniel L. Hood (11)	1989	woman	37	right upper quadrant abdominal pain and right shoulder pain	left hepatic lobe	1,761.25 ml	negative	negative	ovarian mucinous cystadenocarcinoma, total abdominal hysterectomy and bilateral salpingo-oophorectomy	normal
Atsushi Sasaki (12)	1997	Male	42	right abdominal pain and high-grade fever	posterior and medial segment	6.0 cm in diameter; 2.5 cm in diameter	hepatitis B	hepatic cirrhosis	negative	normal
Tohru Ikeda (13)	2003	Man	76	unremarkable	S4 and S7–8 regions of the liver	unknown	type C hepatitis	liver cirrhosis	negative	unknown
M. Ahaouche (14)	2005	man	57	abdominal pain	IVth hepatic segment	6 × 5.5 cm	negative	alcoholic cirrhosis	obesity, diabetes mellitus	12 ng/ml
Udo Rudloff (3)	2005	woman	61	abdominal pain	right lobe of the liver	7 × 7 × 10 cm	fatty liver; no hepatitis	negative	laparoscopic cholecystectomy; coronary artery disease; hypertension	normal
Juergen Bauditz (15)	2006	man	54	no clinical symptoms	segments II and III	7 cm	negative	negative	peripheral arterial disease, hypertension, esophageal reflux, hiatal hernia, and sleep apnea	normal
Chisato Tanahashi (16)	2009	woman	74	unremarkable	left lobe of the liver	10 × 5 cm	negative	negative	negative	unknown
Ryusuke Matsumoto (17)	2012	man	57	jaundice	left hepatic lobe	10 cm	negative	negative	negative	normal
Kyoung-Bun Lee (18)	2014	man	64	resection of a hepatic mass	segment 6	6.0 × 4.0 × 2.2 cm	hepatitis B	hepatic cirrhosis	liver cancer; transarterial embolization (TAE) three times for a 1.4-cm multinodular mass in segment 6 first and percutaneous ethanol injection (PEI) and TAE of a new lesion in segment 6 after 4 years.	36.1ng/mL
Lorenzo Dioscorid (19)	2015	woman	74	dull pain in the right upper quadrant associated with mild anemia	V–VI segments	10 × 7cm	negative	negative	negative	normal
Hans Helmut Dahm (6)	2015	man	68	a tumor of the right lobe of the liver based on ultrasonography that was performed during a routine examination	in segments 7 and 8 of the right liver lobe that adhered to the retroperitoneum.	6 cm in diameter	chronic periportal hepatitis	negative	Insulin dependent type 2 diabetes mellitus and hypertension	unknown
Bita Geramizadeh (20)	2017	woman	64	abdominal pain	right lobe of the liver	20 cm in the greatest diameter	negative	negative	negative	unknown
Meera Balakrishnn (21)	2021	woman	64	abdominal pain associated with nausea	fourth and fifth segments of the liver	11 × 7 cm^2^	negative	negative	hypertension dyslipidemia	normal
Anke H. C. Gielen (22)]	2021	male	77	icterus, general fatigue	segment 8, parts of segments 4 and 5	8cm	negative	negative	hypertension, type 2 diabetes, and prostate cancer	215.5ng/ml

### 3.3 Radiologic characteristics

Hepatocellular carcinoma (HCC) accounts for most liver cancer, and classical HCC appears as arterial phase enhancement on CT followed by washout in the portal phase (23). Cholangiocarcinoma is prevalent in Asia and is divided into intrahepatic, hilar, and distal types. Intrahepatic cholangiocarcinomas show edge enhancement in the arterial phase and a progressive centripetal filling of the fibrous stroma in the delayed phase, with relative low density surrounding it. The imaging features of hilar cholangiocarcinoma are irregular thickening of the hilar bile duct wall, an eccentrically narrowed lumen, and dilation of the upper bile duct. Angiographic findings of distal cholangiocarcinoma included dilatation of the proximal bile duct, confluence of the bile ducts, and enlargement of the gallbladder with normal bile ducts below the stenotic segment. In our case, CT findings of undifferentiated carcinoma with osteoclast-like giant cells of the liver resembled cholangiocarcinoma. We suppose that large intrahepatic masses might contribute to obstruction of bile flow. These were risk factors for the development of cholestasis, bile duct dilatation, and bile duct stones. Lesions showed ring enhancement in the arterial phase and continuous enhancement in the portal phase. These enhancement characteristics suggested that tumors might be abundant in mesenchymal tissue. We summarized the imaging findings of 18 cases of HCC with OCGs reported so far (**Table 3**) (3, 5, 6, 8–22).

**Table 3:** Imaging findings of hepatic tumors with OCGTs.

Study	Imaging findings
Phillip A. Munoz (5)	A liver scan with technetium sulfur colloid revealed diffuse hepatic parenchymal disease with a large focal lesion in the right lobe. A 67-gallium citrate scan suggested a primary liver tumor in addition to cirrhosis.
Hiroyuki Kuwano (8)	Celiac angiography showed a hypervascular tumor shadow, 7.5 × 6.5 cm, in the posterior area of the right lobe of the liver
Salvatore Andreola (9)	A liver echography showed a hypoechoic mass in the right lobe of the liver.
Yutaka Hori (10)	Serial CT scans showed a clustering tumor mass arising from the inferior surface of the liver with low attenuated areas in the mass
Daniel L. Hood (11)	n abdominal computed tomography (CT) scan showed a large hepatic mass
Atsushi Sasaki (12)	Two hepatic tumors were detected in the posterior segment (6.0 cm in diameter) and the medial segment (2.5 cm in diameter), respectively, by abdominal ultrasonography, computed tomography and abdominal angiography.
Tohru Ikeda (13)	unknown
M. Ahaouche (14)	ultrasound and CT scan identified a mass measuring 60 mm in maximum dimension located in the IVth hepatic segment. This nodule had extrahepatic extension and invaded the diaphragm. It was hypervascular and homogeneous.
Udo Rudloff (3)	Magnetic resonance imaging (MRI) of the abdomen confirmed an 8 × 8 × 10 cm mass within the right lobe of the liver involving segments V, VI, and VII. Endoscopic retrograde cholangiopancreatography (ERCP) showed common bile duct (CBD) dilation to 1.3 cm.
Juergen Bauditz (15)	CT demonstrated a 7 cm large inhomogeneous solid liver tumor involving segments II and III.B-mode sonography (HDI 5000, Philips) demonstrated a well-defined, inhomogeneous, cauliflower-like tumor with multiple small calcifications, causing retraction of the liver contour. Within the center of the tumor, a focal nodular hyperplasia (FNH)-like stellar scar was present. Contrast-enhanced sonography demonstrated an inhomogeneous perfused tumor with a large feeding artery heading towards the center of the tumor, radially branching to the periphery.
Chisato Tanahashi (16)	unknown
Ryusuke Matsumoto (17)	Abdominal computed tomography showed a 10-cm left hepatic lobe heterogeneous solid mass with low attenuated areas in the mass, multiple liver metastases and lung metastasis.
Kyoung-Bun Lee (18)	Magnetic resonance imaging showed a 5-cm lobulating soft tissue mass with an internal hemorrhagic component
Lorenzo Dioscorid (19)	the hepatic neoplasm was confirmed and shown to grow from a Riedel’s segment towards the right iliac fossa with a close contiguity with ascending colon and caecum
Hans Helmut Dahm (6)	A computed tomography (CT) scan showed evidence of a malignant liver neoplasm.
Bita Geramizadeh (20)	CT scan showed an enlarged liver with a large mass in the right lobe of the liver with irregular borders and central necrosis, measuring 20 cm in the greatest diameter. A few smaller lesions were also present. Portal vein thrombosis was also identified.
Meera Balakrishnn (21)	Computed tomography (CT) scan showed a heterogeneous focal mass measuring 11x7cm2 occupying the fourth and fifth segments of the liver with areas of necrosis and prominent vessels passing through it. The mass infiltrated the gallbladder wall
Anke H. C. Gielen (22)	A computed tomography (CT) scan showed a liver neoplasm of 6.7 cm in segment 8

### 3.4 Pathological features

There are many controversies surrounding the origin and histogenesis of OCGT. A few studies have suggested that osteoclast-like giant cells were probably neoplastic and epithelial (24). Other studies held the opposite view, that tumors originated from mesenchymal tissue (25, 26). Munoz et al. first reported the case of an osteoclastoma-like giant cell tumor of the liver in 1980. They hypothesized that reticuloendothelial cells were involved in the origin of the tumor (5). Rosai proposed that multinucleated giant cells are always derived from non-epithelial cells with an osteoclastic phenotype and are essentially non-neoplastic, rather than determined by the location of the tumor and the presence of identifiable cancer components in the tumor (2). Based on immunohistochemistry, Sasaki et al. confirmed that the OGC expressed only histiocytic and mesenchymal markers (ACT, AAT, MUR, VIM, and CD68), and they remained negative for epithelial markers (EMA, CK 7, CK 8, and CK 19). Their findings support the view that OCG is non-tumor and non-epithelial (12). In this case, tumors were more likely to be of non-epithelial and mesenchymal origin based on the negative results of EMA and CK (Pan) and the positivity of Vim. The diagnosis of giant cell tumors of bone was unconsidered due to no H3F3A mutation. P53 expression tends to be associated with the differentiation degree of liver tumor cells, especially in poorly differentiated liver tumors, indicating a worse prognosis (27). Hepatic undifferentiated carcinoma is poorly defined from a clinicopathological and molecular perspective. After a full study of 14 cases of primary hepatic undifferentiated carcinoma, high mutation rates of the TERT and TP53 genes and high expression rates of PD-L1 were found, which may be useful biomarkers for potential immunotherapy strategies (28). However, despite a thorough explanation, the patient declined further genetic testing in our case.

### 3.5 Differential diagnosis

There are several subtypes of liver tumors that may be related to multinuclear giant cells and therefore require a differential diagnosis. Hepatocellular carcinoma with syncytial giant cells is a special variety of liver tumor. As the tumor exhibits positive cytokeratin 8 and Hep markers, hepatocellular carcinoma with syncytial giant cells is epithelial and probably arises from hepatocytes (29). Differently, the immunohistological results suggest a different origin of the giant cells, which is more likely to be mesenchymal for our type. As another type of liver tumor containing multinuclear giant cells, sarcomatoid HCC is featured by reactivity for CK 8, ALB, and fibrinogen, as well as for VIM (30). In addition, OCG-associated hepatocellular carcinoma contains two components, including a well-differentiated HCC characterized by partial steatohepatitic morphology and abundant OCG forms mixed with hepatocellular cancer cells (22). The tumor in our case report showed coexistence of undifferentiated carcinoma of the liver and osteoclast-like giant cells, exhibiting negativity for hepatocellular and epithelial markers and positivity for cytokeratin 8/18 (CK8/18).

### 3.6 Treatment

Due to the rarity of the condition, there is not yet a standard therapy for it. It seems that surgery is still the main treatment for this ailment. Masatsugu et al. described radical surgery without any chemotherapy for an undifferentiated liver tumor that achieved good results (31). According to Hood et al., a woman with recurrent OCGT of the liver was treated with 5-fluorouracil and adriamycin, external beam radiation, and radioimmune therapy (IgG, labeled with I-131) (11). The case reported by Kamitani et al. was treated with neoadjuvant chemotherapy before radical hepatectomy. After metastasis occurred two months after surgery, targeted treatment was adopted; however, the patient died five months later (32). In this case, the patient underwent surgery and postoperative adjuvant chemotherapy. The patient is alive after 5-month follow-up.

### 3.7 Prognosis

OCGTs with liver involvement tend to proliferate aggressively. It is apparent that nodal metastasis is the predominant mode of spreading, and the prognosis, even after resection, is usually dismal. Patients survive for weeks to months after surgery for OCGT of the liver. We summarized published cases associated with the prognosis of hepatic tumors with OCGTs from August 1980 to June 2021 on PubMed (**Table 4**) (3, 5, 6, 8–22). As evident from the 18 published cases, most patients died within 3 months of surgery, and only one patient was alive and in good condition.

**Table 4:** Prognosis of hepatic tumor with OCGTs.

Study	Diagnosis	Treatment	Postoperative treatment	Outcome
Phillip A. Munoz (5)	hepatic tumor with OCGTs	no	no	died 32 days later
Hiroyukl Kuwano (8)	HCC with OCGTs	A posterior segmentectomy of the right hepatic lobe with resection of the right diaphragm was performed.	no	metastasis; died four 42 days later
Salvatore Andreola (9)	hepatic tumor with OCGTs	cholecystectomy	no	metastasis; died 20 days later
Yutaka Hori (10)	hepatic tumor with OCGTs	TACE	TACE	died 42 days later
Daniel L Hood (11)	hepatic tumor with OCGTs	resection of the left hepatic lobe and part of the anterior abdominal wall	chemoradiotherapy	metastasis; died three months later
Atsushi Sasaki (12)	Sarcomatoid hepatocellular carcinoma with OCGT	atypical segmentectomy	transcatheteral arterial chemoembolization therapy (TACE)	metastasis, died 28 days later
Tohru Ikeda (13)	sarcomatoid tumor cells with OCGTs	trans-arterial embolization (TAE); Partial hepatectomy	Radiation therapy	metastasis; died one months later
M. Ahaouche (14)	hepatic tumor with osteoclast-like giant cells	lobectomy	no	died 3 months later
Udo Rudloff (3)	hepatic tumor with OCGTs	right hepatic lobectomy	no	metastasis; died three months later
Juergen Bauditz (15)	hepatic tumor with OCGTs	surgical resection of liver segments II and III	chemotherapy	metastasis; alive
Chisato Tanahashi (16)	HCC with OCGTs	left lobectomy	no	died of the disease at 110 days after operation.
Ryusuke Matsumoto (17)	hepatic tumor with OCGTs	no	no	died a few weeks later
Kyoung-Bun Lee (18)	Sarcomatoid hepatocellular carcinoma with OCGT	peripheral segmentectomy of segment 6	no	metastasis
Lorenzo Dioscorid (19)	HCC with OCGTs	hepatic resection of the V-VI segments	no	metastasis; died four months later
Hans Helmut Dahm (6)	HCC, sarcoma with OCGT	atypical segmentectomy	no	metastasis; tumor recurrence
Bita Geramizadeh (20)	hepatic tumor with OCGTs	atypical segmentectomy	no	died two months later
Meera Balakrishnn (21)	hepatic tumor with OCGTs	segmentectomy	chemotherapy	metastasis; died after 108 days
Anke H. C. Gielen (22)	HCC with OCGTs	resection of segment 8 and parts of segment 4 and 5 was performed.	no	alive in a good condition

## 4 Conclusion

Our study provided an in-depth look at imaging observations, treatment modalities, patterns of spread, and clinical outcomes to gain a more comprehensive understanding of this disease. Further studies on undifferentiated hepatic tumors with OGCs are recommended to analyze a suitable therapeutic strategy for this rare condition in the future.

## Data availability statement

The original contributions presented in the study are included in the article/supplementary material. Further inquiries can be directed to the corresponding author.

## Ethics statement

Written informed consent was obtained from the individual(s) for the publication of any potentially identifiable images or data included in this article.

## Author contributions

YD, YW, YZ, and NY conducted the radiological analysis of CT images, XJ conducted the pathological analysis, and YD prepared the manuscript. BW revised the manuscript. All authors listed have made a substantial, direct, and intellectual contribution to the work and approved it for publication.
